# A multiscale computational fluid dynamics approach to simulate the micro-fluidic environment within a tissue engineering scaffold with highly irregular pore geometry

**DOI:** 10.1007/s10237-019-01188-4

**Published:** 2019-06-14

**Authors:** Feihu Zhao, Johanna Melke, Keita Ito, Bert van Rietbergen, Sandra Hofmann

**Affiliations:** 1 Orthopaedic Biomechanics, Department of Biomedical Engineering, Eindhoven University of Technology, PO Box 513, 5600 MB Eindhoven, The Netherlands; 2 Institute for Complex Molecular Systems (ICMS), Eindhoven University of Technology, PO Box 513, 5600 MB Eindhoven, The Netherlands

**Keywords:** Multiscale model, Computational fluid dynamics, Wall shear stress, Homogenization, Tissue engineering scaffold

## Abstract

Mechanical stimulation can regulate cellular behavior, e.g., differentiation, proliferation, matrix production and mineralization. To apply fluid-induced wall shear stress (WSS) on cells, perfusion bioreactors have been commonly used in tissue engineering experiments. The WSS on cells depends on the nature of the micro-fluidic environment within scaffolds under medium perfusion. Simulating the fluidic environment within scaffolds will be important for gaining a better insight into the actual mechanical stimulation on cells in a tissue engineering experiment. However, biomaterial scaffolds used in tissue engineering experiments typically have highly irregular pore geometries. This complexity in scaffold geometry implies high computational costs for simulating the precise fluidic environment within the scaffolds. In this study, we propose a low-computational cost and feasible technique for quantifying the micro-fluidic environment within the scaffolds, which have highly irregular pore geometries. This technique is based on a multiscale computational fluid dynamics approach. It is demonstrated that this approach can capture the WSS distribution in most regions within the scaffold. Importantly, the central process unit time needed to run the model is considerably low.

## Introduction

It is well known that mechanical stimulation can regulate cellular activities. This concept is widely explored in bone tissue engineering (BTE) experiments to stimulate cells to form bone tissue. In such experiments, the mechanical stimulus is often applied by using perfusion bioreactors in which a fluid flow generates a wall shear stress (WSS) to the cell (Bancroft et al. 2003). It has been demonstrated that a WSS in the range of 0.11–10 mPa can stimulate mesenchymal stromal cells (MSCs) to differentiate toward the osteogenic lineage (McCoy and O’Brien 2010), whereas a WSS in a higher range of 0.55–24 mPa can stimulate bone cells to produce mineralized extracellular matrix (ECM) (Vetsch et al. 2017). The resultant WSS on cells is dependent on the flow rate applied to the bioreactors (Guyot et al. 2015, 2016; Zhao et al. 2016). Thus, to determine the flow rate for the bioreactor, computational fluid dynamics (CFD) approaches have been used for calculating the fluidic environment within scaffolds with specific micro-structural geometries (Stops et al. 2010; Papantoniou et al. 2014; Zhao et al. 2018b). In many studies, the scaffolds were idealized with a regular geometry due to the limitations on real geometry meshing and high computational cost (Olivares et al. 2009; Ali and Sen 2018a; Melke et al. 2018; Zhao et al. 2018a). However, a recent study found that the WSS calculated based on idealized scaffolds had considerable differences from the one calculated based on a realistic scaffold geometry (Marin and Lacroix 2015).

To reduce the computational cost, CFD analysis has been applied on one or a few unit-cells of the scaffold (Zhao et al. 2017; Ali and Sen 2018a, b). For scaffolds with a regular pore geometry, it usually suffices to analyze only a small unit-cell of the sample, since the results for the full sample can be obtained by repetition of the unit-cell results (Marin and Lacroix 2015; Hendrikson et al. 2017). However, in many tissue engineering experiments, the scaffolds have a highly irregular pore geometry, e.g., silk fibroin (SF) scaffolds (Melke et al. 2016) and collagen-GAG scaffolds (Mccoy et al. 2012), that cannot be well represented by a repetitive unit-cell. Performing a CFD analysis for the complete scaffold typically is inhibited by the high computational costs involved and by challenges in creating the complex meshes. The CFD analysis in such cases is typically limited to analyzing one or more relatively small representative volume elements (RVEs) (Sandino et al. 2008; Stops et al. 2010; Zhao et al. 2017). The accuracy of such analyses will depend on many factors, e.g., homogeneity of the scaffold, prescribed boundary/loading conditions (Hu et al. 2018). Maes et al. (2012) tested the accuracy of WSS calculations by CFD, and found that RVE size had a distinct influence on the calculated WSS under the idealized boundary conditions. They also found that applying idealized boundary/loading conditions could not capture the real WSS distribution within the global scaffold under perfusion flow (Maes et al. 2012).

A potential way to improve the accuracy of the WSS calculations without having to perform the CFD for the full scaffold would be to use a multiscale approach. With a multiscale approach, one or more micro-structural RVEs obtained from the scaffold could be homogenized first using a CFD analysis to obtain their permeability, which then is assigned to a macro-structural model representing the full scaffold. The macro-structural model can be used to calculate the boundary conditions for the micro-structural model. Afterward, a realistic WSS within RVEs can be calculated using a second CFD analysis. To the best of our knowledge, this approach has not been used yet for the analysis of WSS in tissue engineering scaffolds. Thus, it is unclear to what extent this technique can improve the accuracy and if the computational costs are in an acceptable range to avoid the need for high performance computing (HPC) facilities.

In this study, we aim to develop a multiscale CFD approach for quantifying the micro-fluidic environment in highly irregular scaffolds, and to test its accuracy and feasibility. To test the accuracy, CFD results obtained for a micro-structural model that represents a complete but small scaffold are compared to those obtained from the multiscale approach. Such comparison is based on the WSS in a region of interest (ROI) of the same small scaffold. To test the feasibility for modeling realistic sample sizes, a multiscale analysis based on multiple micro-structural ROIs of a larger scaffold is used to evaluate the homogeneity of the WSS in a complex scaffold under medium perfusion.

## Materials and methods

### Multiscale computational framework

**Fig. 1:**
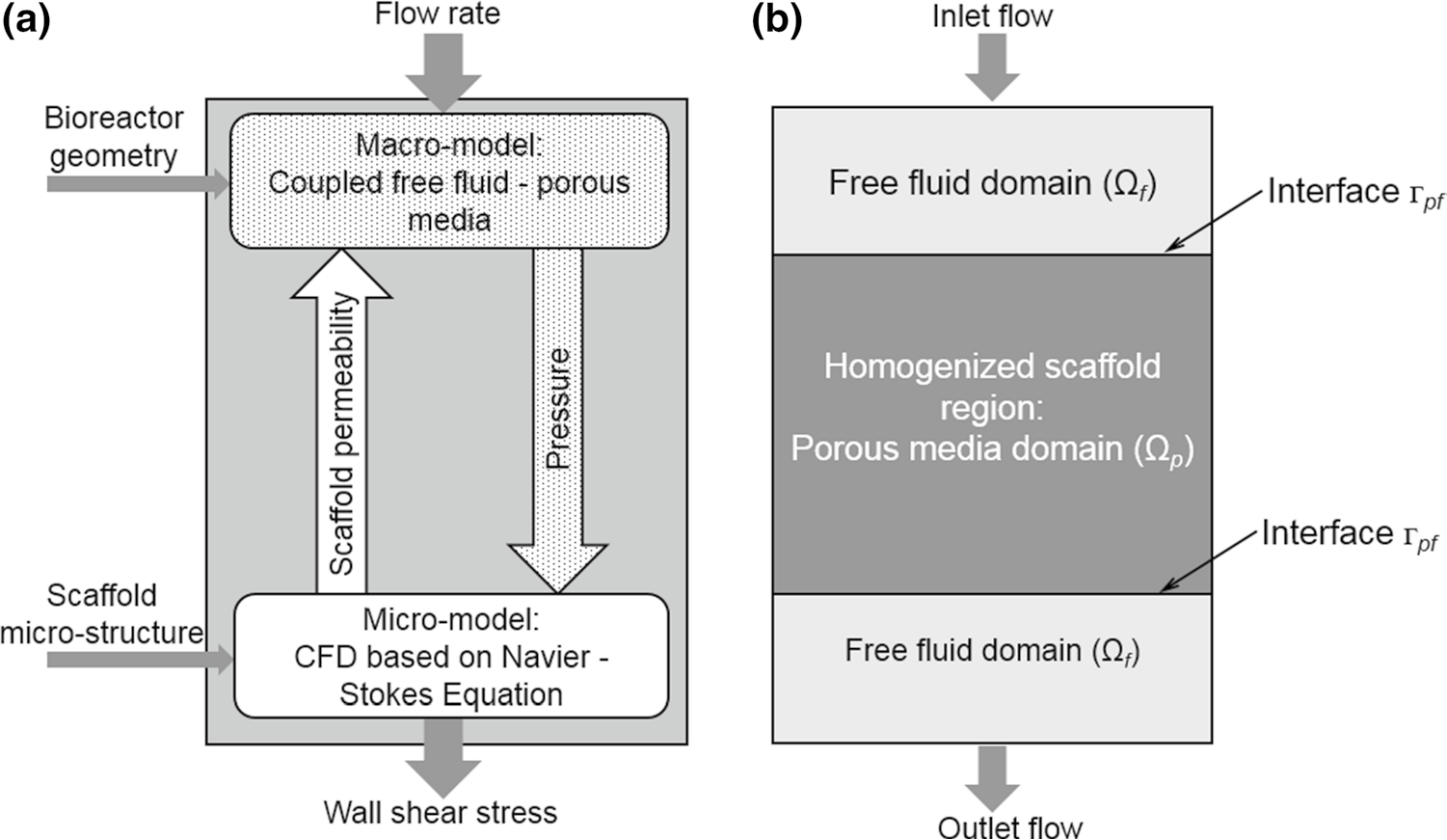
**a** Overview of the multiscale CFD approach for calculating the WSS within RVE of a scaffold with highly irregular pore geometries; **b** schematic representation of the macro-structural model

The multiscale framework developed here consisted of (1) a macro-structural model representing the full scaffold and the perfusion bioreactor channel, (2) a micro-structural model representing the scaffold micro-structural geometry in detail (Fig. 1a). The micro-model contained one or more RVEs, depending on scaffold homogeneity and size.

First, a CFD analysis was performed at the micro-level to calculate the micro-structural (i.e., scaffold RVE) permeability. This permeability then was then assigned to the macro-structural model (Fig. 1a) which then was used for calculating the pressure drop over the scaffold, depending on the assigned permeability and the applied flow rate. Afterward, the calculated pressure obtained from the macro-model was assigned as a boundary/loading condition to the micro-structural model (Fig. 1a). Finally, the micro-structural model was solved again to calculate the cell-level WSS under the defined specific boundary/loading conditions. In the following paragraphs, each of these steps would be presented in detail.

#### Micro-level: scaffold permeability calculation

The fluid domain geometry of a RVE micro-structural model was generated from micro-CT images of the scaffold by Boolean operations in FreeCAD (Riegel et al. 2016). After the mesh sensitivity analysis (“Appendix 1”), we meshed the fluid domain by tetrahedral elements (element type: FC3D4) with global maximum and minimum sizes of 50 µm and 5 µm, respectively. The actual mesh size was controlled by the local curvature of the scaffold surfaces with a maximum deviation factor (ratio between chordal deviation and element size) of 0.1.

Equation (1) derived from Darcy’s law was used for calculating the permeability of the RVE:

1 $$Q = \frac{\kappa A}{\mu } \cdot \frac{{\Delta p}}{H}$$

where Δ*p* is the pressure drop over the scaffold height *H*, Δ*p* is calculated from the CFD model, *Q* is the prescribed flow rate, *A* is the cross-sectional area to flow, *µ* is the dynamic viscosity of the culture medium (Dulbecco’s Modified Eagle medium supplemented with 10% FBS) with a value of 1.0 mPa s (Maisonneuve et al. 2013), and *κ* is the permeability.

The CFD models were solved by a finite volume method (FVM) using ANSYS CFX (ANSYS Inc., PA, USA) under the convergence criteria of root-mean-square residual of the mass and momentum < 10^−4^.

#### Macro-level: pressure calculation

The macro-structural model representing the full scaffold and perfusion bioreactor channel was used for calculating the pressure gradient within the scaffold under fluid flow as described in Fig. 1a. In this macro-structural model, the scaffold region was homogenized and assigned a permeability that had been calculated from one or more RVE micro-structural models (Fig. 1a). In this multiscale framework, the RVE of the micro-structural models covered the full height of the scaffold, avoiding the influence of heterogeneity in height direction of the macro-model in the pressure calculation. The region where the scaffold was placed was defined as homogeneous porous media (denoted as Ω_p_ in Fig. 1b), following Darcy’s equation (Eq. 2):

2 $$\left\{ {\begin{array}{*{20}l} {\nabla \cdot \left\langle {\mathbf{v}} \right\rangle = 0} \hfill \\ {\frac{\mu }{\kappa }\left\langle {\mathbf{v}} \right\rangle + \nabla \left\langle p \right\rangle = 0} \hfill \\ \end{array} } \right.\quad {\text{in}}\,\varOmega_{\text{p}}$$

where 〈**v〉** is the Darcy velocity (average fluid velocity), 〈*p*〉 is the average pressure, and Ω_p_ represents the homogeneous porous media domain.

The other regions within the bioreactor channel were defined as free fluid (incompressible, Newtonian laminar flow), which followed the Navier–Stokes equation (Eq. 3):

3 $$\left\{ {\begin{array}{*{20}l} {\nabla \cdot {\mathbf{v}} = 0} \hfill \\ {\frac{{\partial {\mathbf{v}}}}{\partial t} + {\mathbf{v}} \cdot \nabla {\mathbf{v}} = - \nabla p + \mu \nabla^{2} {\mathbf{v}}} \hfill \\ \end{array} } \right.\quad {\text{in}}\,\varOmega_{\text{f}}$$

where **v** is the fluid velocity vector, and Ω_f_ represents the free fluid domain.

On the boundaries, the condition of continuity of mass flux is defined as Eq. (4):

4 $$\rho \left\langle {\mathbf{v}} \right\rangle \cdot {\mathbf{n}}_{\text{p}} \left| {_{{\varGamma_{\text{p}} }} } \right. = 0;\quad \rho {\mathbf{v}} \cdot {\mathbf{n}}_{\text{f}} \left| {_{{\varGamma_{\text{f}} }} } \right. = 0;\quad \rho \left\langle {\mathbf{v}} \right\rangle \cdot {\mathbf{n}}_{\text{p}} \left| {_{{\varGamma_{{\text{pf}}} }} } \right. = \rho {\mathbf{v}} \cdot {\mathbf{n}}_{\text{f}} \left| {_{{\varGamma_{{\text{pf}}} }} } \right.$$

where *ρ* is the density of the medium (1000 kg/m^3^), г_p_ and г_f_ define the exclusive boundary of Ω_p_ and Ω_f_, respectively, г_pf_ the interface between Ω_p_ and Ω_f_, and **n**_p_ and **n**_f_ are the vector normal to г_p_ and г_f_, respectively (Fig. 1b).

#### Micro-level: WSS calculation

The same micro-structural models as the ones originally used to calculate the permeability calculation were also used for calculating the WSS distribution on the scaffold surfaces. The fluid pressure derived from the macro-model was prescribed at the inlet surface of the micro-model. The side surfaces were defined as symmetric boundaries with the fluid velocity *v* according to Eq. (5):

5 $$\left\{ {\begin{array}{*{20}l} {v_{j} = 0} \hfill \\ {\frac{{\partial v_{i} }}{{\partial x_{j} }} = 0} \hfill \\ \end{array} } \right.$$

with *j* being the direction perpendicular to the cutting surface.

The WSS on the scaffold surface (Г_S_) then was calculated according to:

6 $$\tau_{ij} = \mu \left. {\left( {\frac{{\partial v_{i} }}{{\partial x_{j} }} + \frac{{\partial v_{j} }}{{\partial x_{i} }}} \right)} \right|_{{x_{i} \in\Gamma _{\text{S}} }}$$

where, *x*_*i*_ (or *x*_*j*_) is the *i*th (or *j*th) spatial coordinates.

### Material

In this study, this multiscale CFD framework was applied on a silk fibroin (SF) scaffold, which was used widely in tissue engineering experiments, and had highly irregular pore geometries (Melke et al. 2016). The SF scaffold was fabricated using the same approach as described in previous study (Melke et al. 2018). To obtain the scaffold geometry, micro-computed tomography (micro-CT) scanning was carried out on a dry and empty SF scaffold in a dry environment using a micro-CT 80 scanner (Scanco Medical AG, Brüttisellen, Switzerland) at a nominal isotropic resolution of 10 μm. The energy level was set to 45 kVp, intensity to 177 µA, 300 ms integration time and two-fold frame averaging. A 3D constrained Gaussian filter (sigma = 0.8, support = 1 voxel) was applied.

**Fig. 2:**
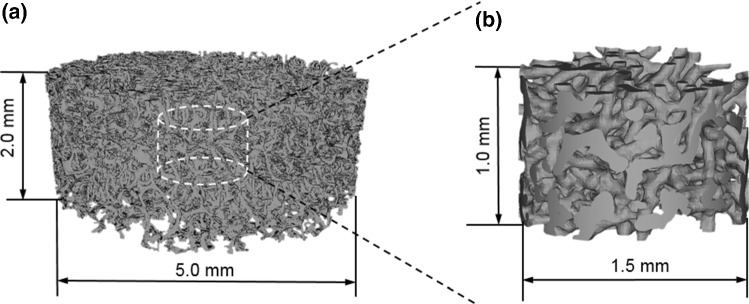
**a** Full SF scaffold with dimensions typically used for tissue engineering applications, **b** a sub-section from the same scaffold used in the accuracy study

The micro-CT images of the scaffold were segmented with a threshold of 1087–1151 Hounsfield units using MIMICS (Materialise NV, Leuven, Belgium) for extracting the air. To remove the noise, the whole scaffold geometry was smoothed for 4 iterations with a smoothing factor of 0.4. The whole scaffold size was 5 mm in diameter and 2 mm in height, which was considered typical for tissue engineering applications (Fig. 2a). Considering the high computational cost for validation study (Sect. 2.3), from this full scaffold, a smaller central cylindrical region of 1.5 mm in diameter and 1.0 mm in height was selected to validate this multiscale CFD approach (Fig. 2b).

### Validation study

**Fig. 3:**
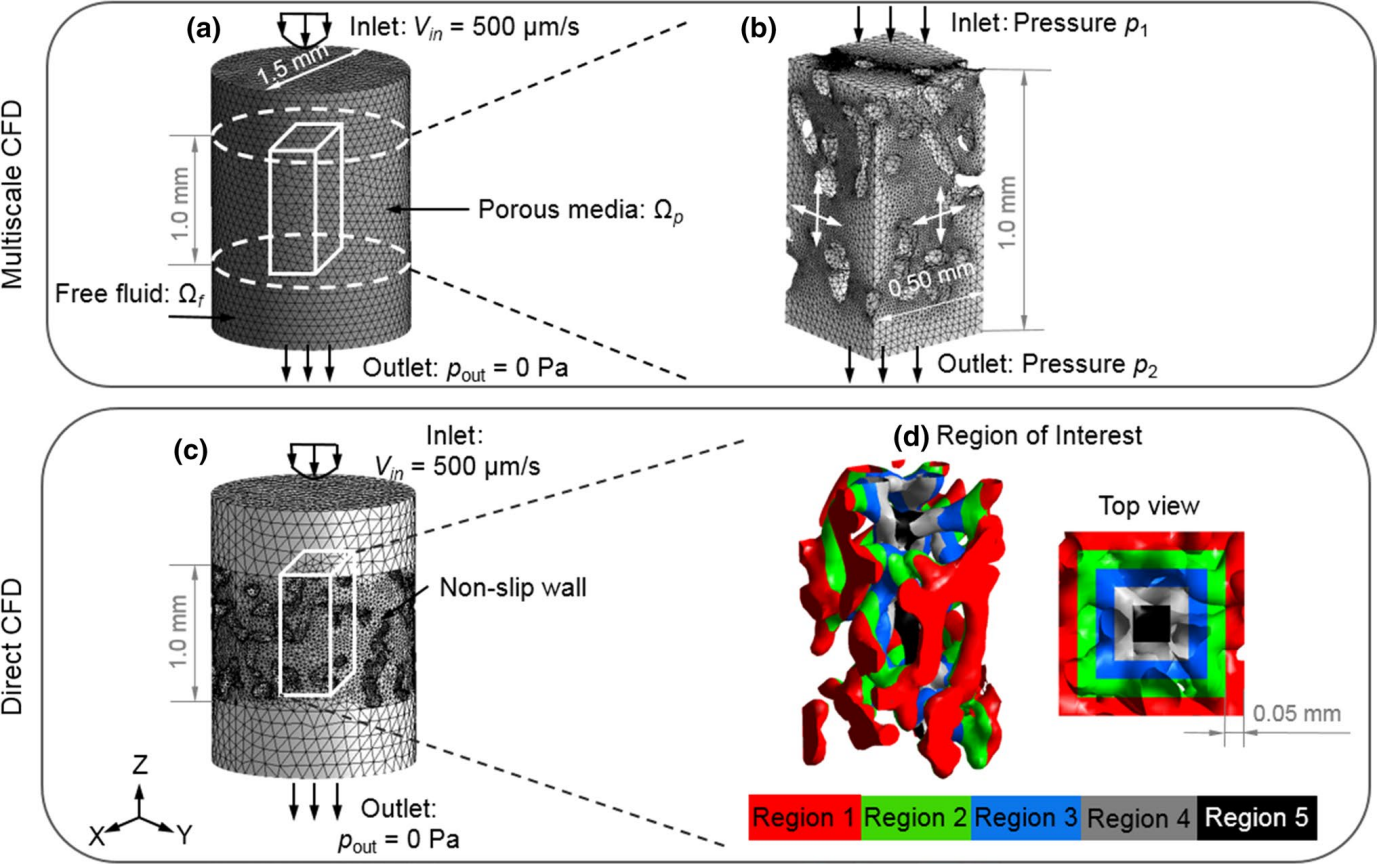
Mesh and boundary conditions of the **a**, **b** multiscale CFD model, which involves **a** a free fluid—porous media model (macro-structure) and **b** CFD model of RVE section (micro-structure); **c**, **d** direct CFD model based on the full scaffold, **d** ROI in the direct CFD model: average WSS is calculated in 5 sub-regions (regions 1–5) separately, each region has a width of 0.05 mm

To examine the accuracy of the multiscale CFD approach, we compared the WSS distribution calculated by the multiscale CFD approach to those calculated from a micro-structural CFD analysis that comprised the full scaffold. Due to the excessively high computational cost of performing the micro-structural CFD model for the large scaffold, the validation was performed on the small whole scaffold (Fig. 3b). For this scaffold, a single RVE of 0.5 × 0.5 × 1.0 mm in size, which could cover at least 1 complete pore was defined at the center of the scaffold, considering the results from RVE size sensitivity analysis (“Appendix 2”). For this RVE, the permeability was calculated, which was then assigned to the macro-model. For the macro-model, a fluid velocity of 500 µm/s was prescribed at the inlet while the four side surfaces were defined as non-slip walls, where the fluid had zero velocity relative to the boundary (Fig. 3b). The outlet was prescribed with a relative pressure of 0 Pa. By assigning the fluid pressure to the micro-structural model and solving the micro-structural model, the WSS within RVE was calculated.

In a direct CFD approach, Eq. (3) was solved for the small-sized scaffold (diameter = 1.5 mm, height = 1.0 mm in Fig. 2b). The CFD models were run using a computer with 16 GB RAM and 8 cores (CPU: Intel i7-6700). Finally, a histogram of WSS distribution from multiscale CFD and direct CFD approaches was counted, and the correlation of the WSS distribution results in RVE (by multiscale CFD model) and ROI (by direct CFD model) was characterized with a Pearson correlation coefficient. Furthermore, the symmetric boundary conditions in the multiscale CFD model might potentially affect the WSS results. Therefore, the percentage error between the WSS computed by multiscale CFD and direct CFD approaches was calculated in 5 sub-regions of the ROI/RVE (i.e., region 1–5 in Fig. 3d).

### Feasibility of the multiscale CFD approach for the analysis of large scaffolds

To test the feasibility for upscaling to larger scaffolds and to investigate the effect of pore inhomogeneity on local WSS, the multiscale CFD approach was applied to model the full SF scaffold (Fig. 2a).

**Fig. 4:**
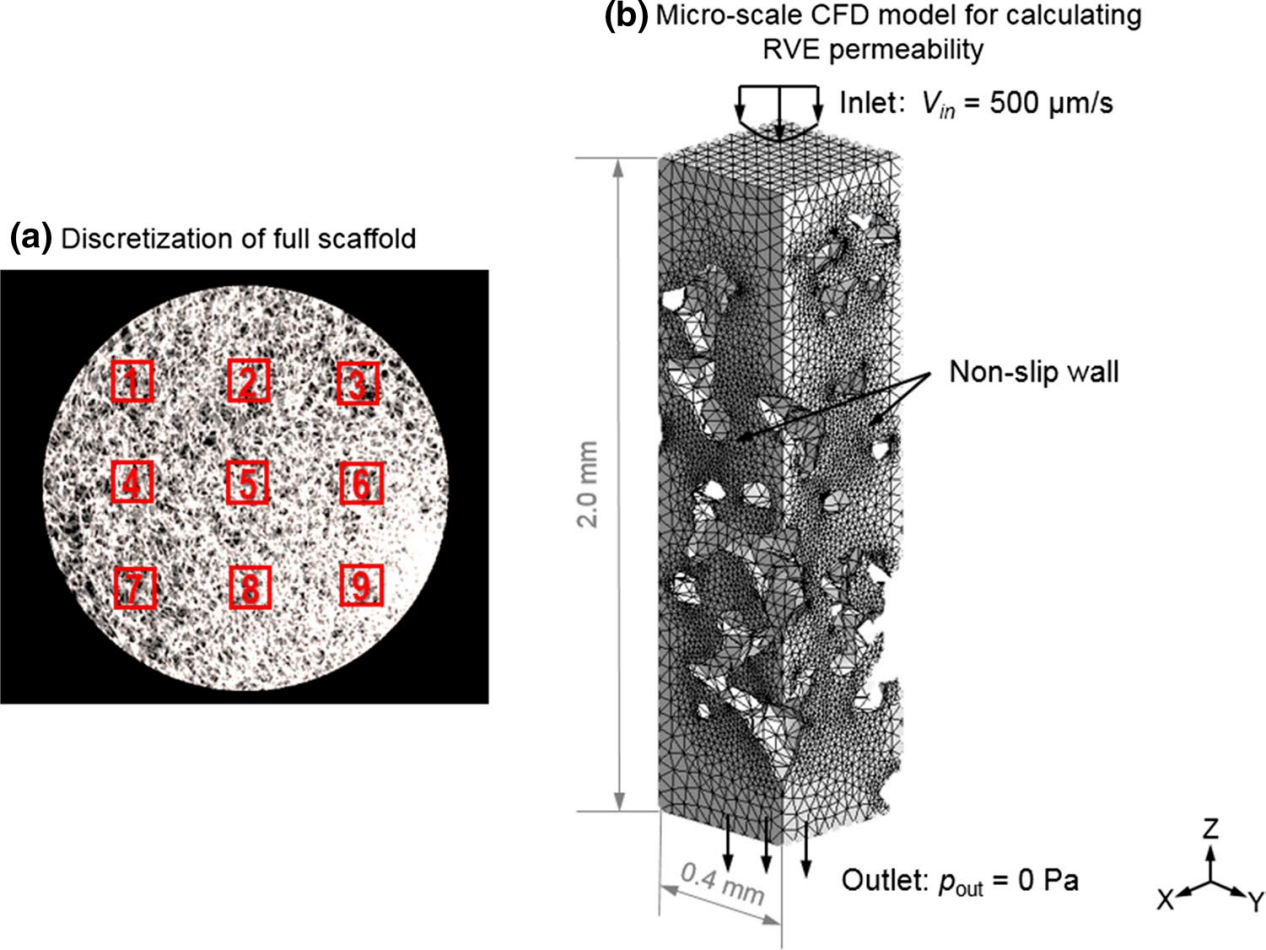
**a** Full SF scaffold is discretized into 9 isometric RVE sections, **b** the fluid domain and the boundary conditions of the CFD model (micro-level) for calculating the permeability of one example RVE section

For this scaffold, 9 RVEs were selected as shown in Fig. 4a. To cover the full specimen by 9 isometric RVEs, each RVE was defined with a dimension of 0.4 × 0.4 × 2.0 mm, covering the full specimen height (Fig. 4b). The approach described in Sect. 2.1 was used for calculating the permeability of each RVE section. In permeability calculation, an inlet fluid velocity of 500 µm/s was prescribed at the inlet surface of the RVE (Fig. 4b) same as that in Sect. 2.3.

**Fig. 5:**
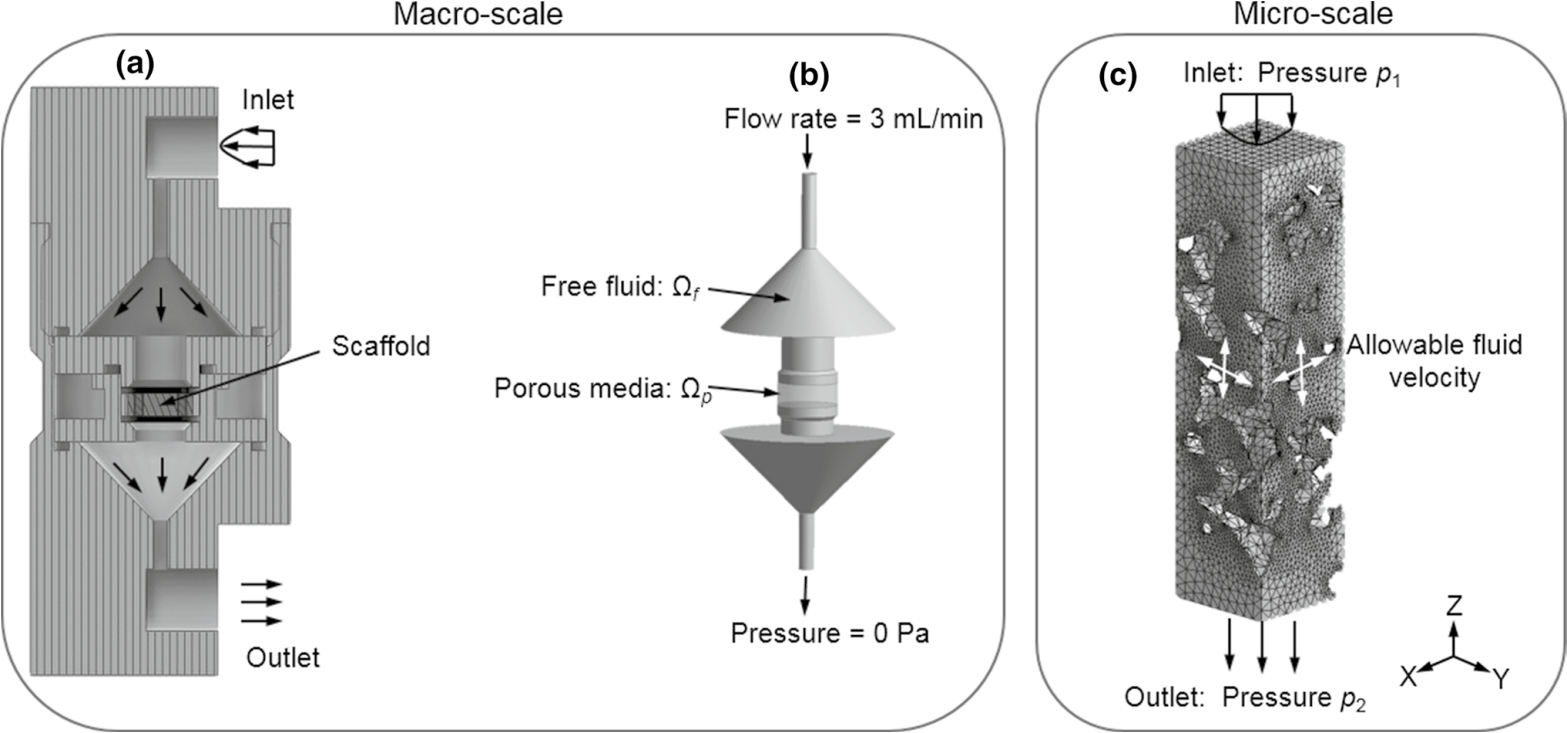
At macro-scale: **a** schematic image of a flow perfusion bioreactor system (Zermatten et al. 2014; Vetsch et al. 2017), **b** fluid domain of the bioreactor system, which includes free fluid domain (Ω_f_) and porous media domain (Ω_p_); at micro-scale: fluid domains and boundary conditions of the micro-CFD model for calculating WSS within one example RVE

The flow perfusion bioreactor design and dimensions were taken from previous studies (Zermatten et al. 2014; Vetsch et al. 2017) (Fig. 5a), and the model geometry was meshed by 343,911 tetrahedral elements. At the inlet of the model, a constant flow rate of 3 mL/min was prescribed, which was considered to be within a range preferable for bone tissue mineralization in vitro (Zhao et al. 2018b). In this macro-model, the scaffold region was modeled as a porous media (Ω_p_ in Fig. 7b), which followed Darcy’s law (Eq. 2). Its permeability was calculated by averaging the permeability values of the 9 RVEs. Although the actual permeability was not homogeneous throughout the scaffold, modeling it as homogeneous in the macro-model was sufficient as the macro-model was used only for calculating the pressure boundary conditions for the micro-models (analysis in “Appendix 3”). The other regions within the bioreactor were modeled as free fluid (Ω_f_ in Fig. 5b), following the Navier–Stokes equation (Eq. 3) as described in Sect. 2.1.

The same RVE models used for the permeability calculation were also used for calculating the WSS distribution on RVEs surfaces. Results presented here were only for three representative RVEs (RVE 1, 5 and 9 as shown in Fig. 4a). The fluid pressure derived from the macro-model was prescribed on the inlet surface. The side surfaces were defined as symmetric boundaries with the fluid velocity *v* according to Eq. (5). The multiscale CFD model was also run on a computer with 16 GB RAM and 8 cores (CPU: Intel i7-6700).

## Results

### Accuracy of the multiscale CFD model

The permeability of the homogenized scaffold domain (porous media Ω_p_) was 7.47 × 10^−10^ m^2^. When assigning this permeability to the macro-model, a pressure drop over the scaffold height (Δ*p*) of 0.70 Pa was obtained for the prescribed fluid velocity.

**Fig. 6:**
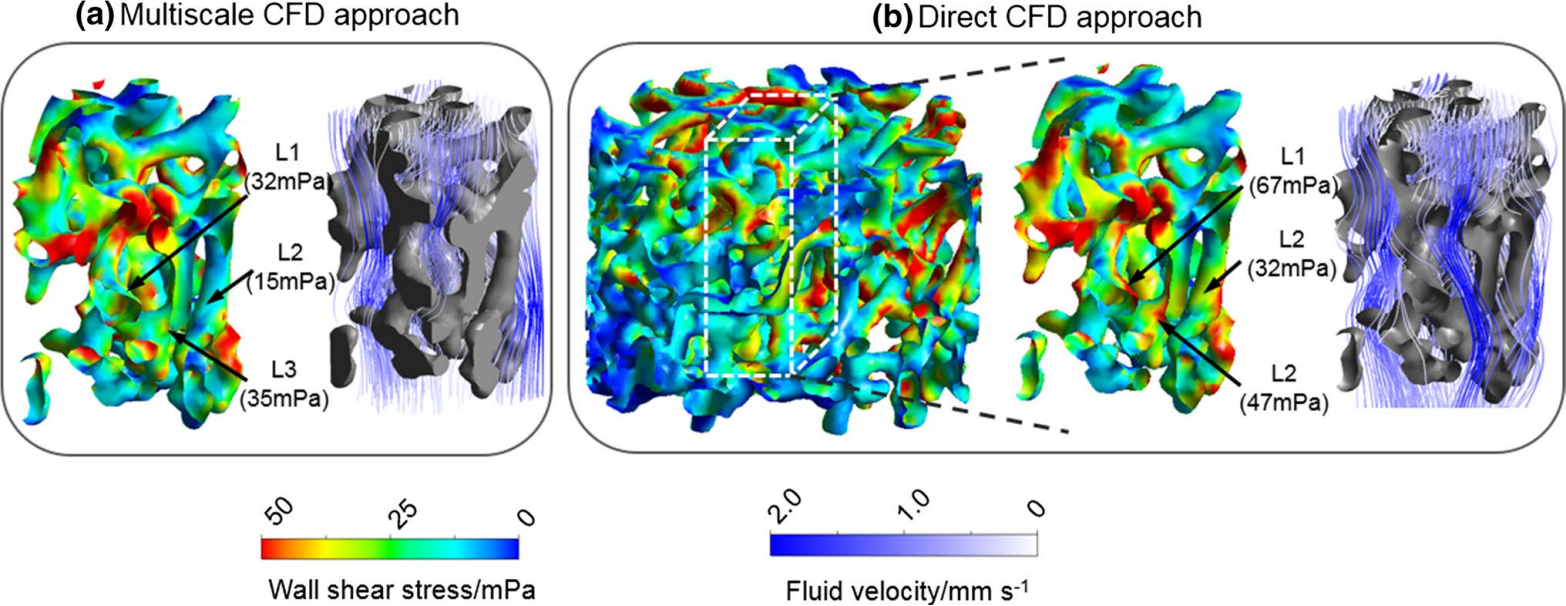
WSS and fluid velocity distribution within the RVE section, which are calculated from **a** multiscale CFD model and **b** direct CFD model of the full scaffold, respectively. L1, L2 and L3 are the locations, where the WSS has distinctly different values between multiscale CFD and direct CFD approaches

**Fig. 7:**
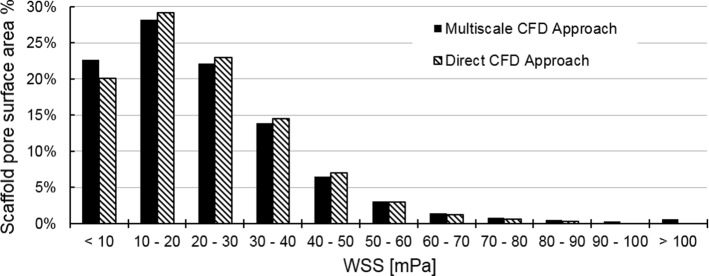
WSS distribution within RVE section and ROI, which have been calculated by either the multiscale CFD approach (black) or the direct CFD approach (shaded), respectively

**Fig. 8:**
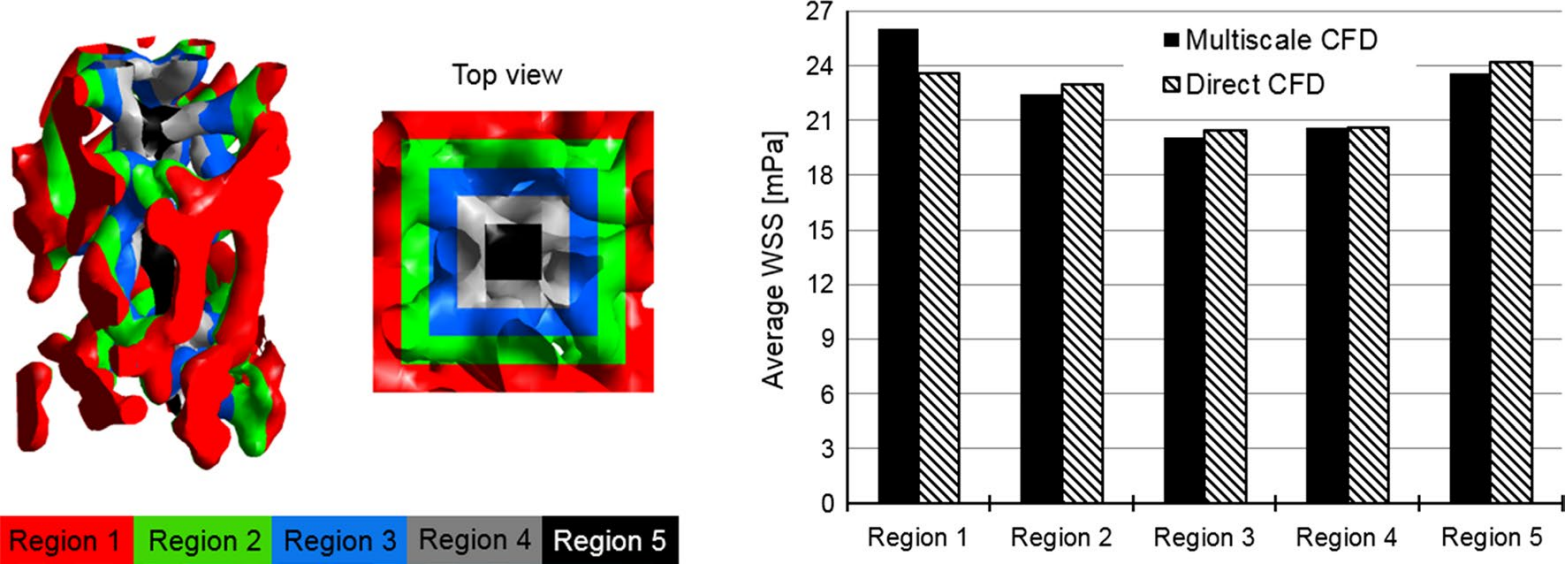
Average WSS that is calculated from 5 regions (from outer boundaries toward inner area: region 1–5) by a multiscale CFD approach (black) and a direct CFD approach (shaded), respectively. The Pearson correlation coefficient between multiscale CFD and direct CFD approaches is 0.86 on the WSS distribution

Under this fluid pressure, the WSS distribution within the RVE scaffold section was calculated (Fig. 6a). For comparison, the WSS distribution within RVE/ROI calculated by multiscale CFD and direct CFD approaches, respectively, is presented in a histogram in Fig. 7. The Pearson correlation coefficient between the WSS distributions computed by multiscale CFD and direct CFD approaches was 0.86. By comparing the average WSS calculated by two approaches at different sub-regions (i.e., region 1–5 in Fig. 8), it was found that the largest difference of the average WSS between two approaches was in region 1 (close to the outer boundaries) with a percent error of 10.5%. In the inner regions (region 2–5 in Fig. 8), the percentage error of the average WSS between two approaches was smaller, i.e., 2.2%, 1.7%, 0.05% and 2.3% for region 2, 3, 4 and 5, respectively. By comparing the WSS at specific locations calculated by the two approaches (Fig. 6), it was found that the largest differences (i.e., up to 1.1-folds difference) in WSS were found close to the boundaries as pointed out in Fig. 6 (L1–L3). Nevertheless, the middle regions within the RVE had a similar WSS distribution between two cases as shown in Figs. 6 and 8.

The central process unit (CPU) time spent on solving the direct CFD model on the full scaffold (i.e., diameter = 1.5 mm, height = 1.0 mm in Fig. 2b) was 20 min, while the CPU time needed for the multiscale approach was 5.8 s (macro-structural model in Fig. 3a) and 1.6 min (micro-structural model in Fig. 3b).

### Feasibility of applying a multiscale CFD model on a large scaffold

This multiscale CFD framework was successfully applied on a large SF scaffold (diameter = 5 mm, height = 2 mm) with a highly irregular pore geometry to simulate the micro-fluidic environment within the scaffold. Low CPU time was needed to run this multiscale CFD model based on the large-sized and irregular scaffold. For instance, merely 2.5 min of CPU time was needed for running the macro CFD model of the perfusion bioreactor system (model in Fig. 5a). For the micro-structural CFD models (e.g., 9 RVEs in Fig. 4a), 1.5–6.0 min of CPU time was needed for each sub-CFD model, depending on the volume of different liquid-phase RVE sections (i.e., counterparts of scaffold solid RVEs).

**Fig. 9:**
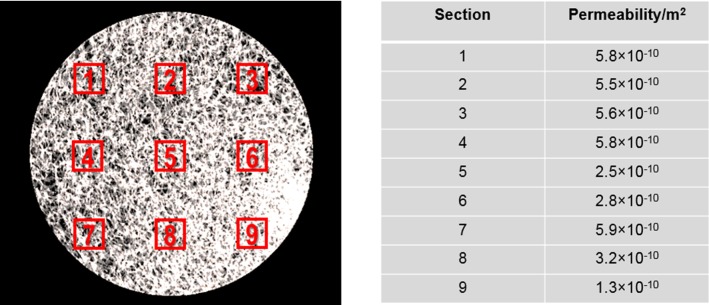
Permeability of 9 different RVE sections, the average permeability of the homogenized whole scaffold is 4.27 × 10^−10^ m^2^

The permeability of 9 RVE sections from the global scaffold was calculated (Fig. 9) and resulted in an average permeability of 4.27 × 10^−10^ m^2^ that was used as the permeability of the porous media domain in the global CFD model. Using this model, a pressure drop of 2.66 Pa was calculated from the top (3.28 Pa) to bottom (0.62 Pa) surface of the scaffold for a flow rate of 3 mL/min (“Appendix 4”). Since the RVE dimension in longitudinal direction (or Z-direction in Fig. 5b) was completely preserved, the pressures of *p*_1_ = 3.28 Pa and *p*_2_ = 0.62 Pa were defined on the upper and bottom surfaces, respectively (Fig. 5b).

**Fig. 10:**
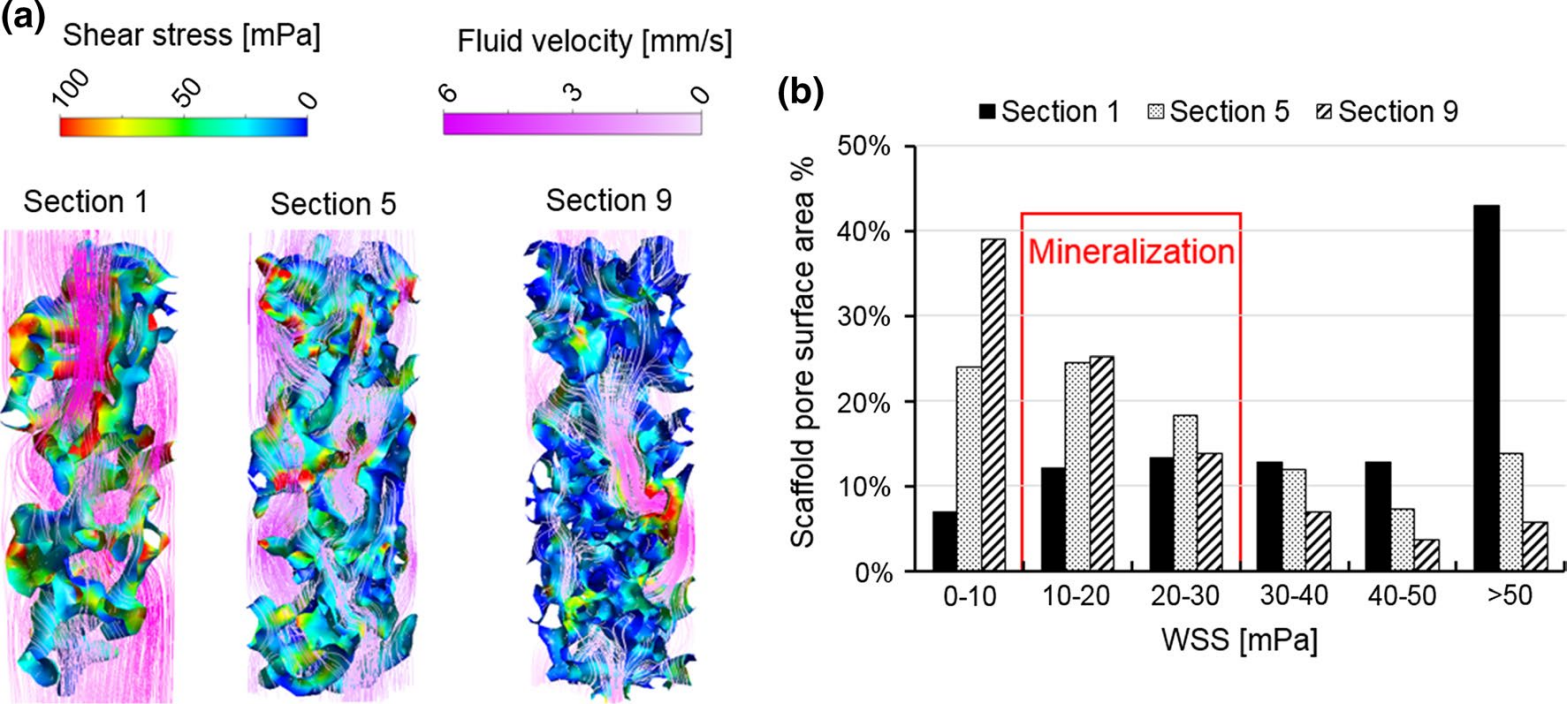
**a** WSS and fluid velocity distribution within three different RVE Sections (1, 5 and 9); **b** scaffold surface area fractions that will undergo different ranges of WSS and will stimulate different cell behavior

After solving the local CFD models, the WSS and fluid velocity distributions in RVE 1, 5 and 9 are shown in Fig. 10a as examples. The highest fluid velocity and WSS are found in Sect. 1, which had the highest permeability of these three sections.

## Discussion

To enable the simulation of the micro-fluidic environment within a tissue engineering scaffold with a highly irregular pore geometry, a multiscale CFD framework with low-computational cost but high accuracy was developed in this study. This multiscale CFD model was verified by comparing it to the direct CFD model on the calculated WSS within a small scaffold volume. To demonstrate its feasibility of modeling the large-sized scaffold with irregular pores geometry, in particular with low requirement on the computing resource, we have successfully applied it in simulating the micro-fluidic environment within a realistic-sized scaffold specimen, which has a diameter of 5 mm, height of 2 mm and highly irregular pore geometry.

One limitation of this multiscale CFD model was the boundary conditions defined on the micro-scale model. As illustrated in Figs. 3b and 5c, a symmetric boundary condition was prescribed on the four side surfaces of the RVE. This prevented any lateral flow (i.e., flow between RVEs), which might occur in reality. This would potentially have an effect on the resultant WSS. However, these effects would occur mainly near the boundaries as can be seen in Fig. 6 (e.g., WSS at L1–L3). When comparing the multiscale CFD with the direct CFD approach, the largest average WSS errors was in the region close to the symmetric boundaries. Nevertheless, even in these regions the largest error in the calculated WSS was considered low (< 10.5%). Moreover, having analyzed the WSS in all the regions, the multiscale CFD and the direct CFD approaches showed a Pearson correlation coefficient of 0.86, which was a highly positive correlation according to (Mukaka 2012). This indicated that the multiscale CFD model could well capture the real WSS distribution within the RVE. A similar study simulating the micro-fluidic environment within SF scaffolds has applied another strategy (i.e., changing mesh density in ROI of the direct CFD model) to avoid additional boundary/loading condition prescription across the scales (Zermatten et al. 2014). For modeling the highly irregular scaffold, in particular with a realistic size, a much coarser mesh had to be applied on the non-ROI. As the non-ROI and ROI were in the same CFD model, this technique of changing mesh density would potentially affect the micro-fluidic environment within the ROI. It was found that the average WSS would change by 11% when the scaffold was meshed by 1.13 × 10^8^ and 2.33 × 10^7^ elements, respectively (Zermatten et al. 2014). In our multiscale CFD model, such mesh density issue was avoided, as the volume/size of RVEs and their mesh density could be adjusted accordingly. For instance, to improve the accuracy and not to increase the computational cost, we might discretize the large full scaffold into more RVEs with smaller volume/size, which consequently can be meshed with a more refined meshing density. However, to be representative of the porous structure, the RVE should cover the dimensions of at least one complete pore, meaning that the RVE size needed to be larger than the average pore diameter. Considering this and the computational limit for the direct CFD model, we used only one cut-out RVE from the full scaffold sub-section in our validation study. Nevertheless, the results demonstrated that even with one cut-out RVE, the multiscale CFD model showed a high agreement with the direct CFD model (Pearson correlation coefficient = 0.86). Furthermore, the calculated WSS also depended on the resolution of micro-CT for scanning the scaffold. If the strut size was smaller than the micro-CT resolution, such strut geometry would be missed by micro-CT scanning, comparing to the other high-resolution approaches (e.g., transmission electron microscope and scanning electron microscope) (Gashti et al. 2012). In this case, CFD model was not able to capture the real WSS on these detailed small struts.

Another factor that could influence the accuracy of the multiscale CFD model is the inhomogeneity of the scaffold. If the scaffold pore geometry was highly inhomogeneous, the calculated WSS would be less accurate if the scaffold was discretized into fewer RVEs in scaffold’s homogenization. However, this potential limitation could be addressed by discretizing the global scaffold into more RVEs in scaffold homogenization.

This particular type of SF scaffold has previously been used for various tissue engineering applications (e.g., bone, tendon/ligaments, nerves) (Ding et al. 2014; Melke et al. 2016; Maghdouri-White et al. 2018; Nune et al. 2019). For bone tissue engineering in vitro, different WSS ranges have been found to stimulate seeded cells to produce mineralized ECM, e.g., 0.55–24 mPa (Vetsch et al. 2017), 5–15 mPa (Li et al. 2009) and 10–30 mPa (Sikavitsas et al. 2003). For example, if the WSS range of 10–30 mPa was applied to the WSS within RVE Sects. 1, 5 and 9, it was calculated that 25.3%, 43.0% and 39.0% of the scaffold surface area was likely to undergo the WSS that would stimulate cells to produce mineralized ECM (Fig. 10b). In RVE Sect. 1, a larger scaffold surface area underwent the WSS beyond the range of 10–30 mPa, e.g., 43% of the surface area in RVE Sect. 1 was exposed to WSS higher than 50 mPa. The WSS in RVE Sect. 9 was low with 39% of the surface area exposed to WSS below 10 mPa. Thus, it was likely that the mineralized bone tissue growth within this global SF scaffold would be inhomogeneous due to the variation of the local mechanical stimulation at different regions. This has been observed experimentally in a recent study, in which WSS is applied for bone tissue mineralization in vitro by a spinner flask bioreactor (Melke et al. 2018). It was found that the variation of WSS distribution within scaffold was associated with inhomogeneous mineralized tissue distribution (Melke et al. 2018).

The multiscale CFD model resulted in a substantially low CPU time. In this study, we used a computer with 16 GB RAM and 8 cores (CPU: Intel i7-6700) to run the multiscale CFD model. For running the macro-structural CFD model of the perfusion bioreactor system, merely 2.5 min of CPU time was used. For the micro-structural CFD models that were based on the RVEs of the SF scaffold, 1.5–6.0 min of CPU time was needed for each sub-CFD model, depending on the volume of different RVE sections in liquid phase. The computational cost did not exclusively mean the CPU time for running the CFD model, it also reflected on the computing resources (i.e., HPC clusters) that could achieve model geometry reconstruction and meshing. In a previous CFD study based on the similar setup of the bioreactor systems, it was declared that the requirement of high computational cost was a drawback (Zermatten et al. 2014). A trade-off meshing strategy (i.e., varying mesh density based on ROIs) had to be employed for successfully running the CFD model (Zermatten et al. 2014). However, full scaffold geometry reconstruction and meshing also have high requirements on the computing resources. We also attempted to apply the direct CFD model on the large entire scaffold (i.e., the one in Fig. 2a). However, it was even not feasible to reconstruct and mesh the geometry of the large entire scaffold for direct CFD approach on a computer with 16 GB RAM and 8 cores (CPU: Intel i7-6700). Nevertheless, with our multiscale approach, the potential geometry reconstruction and meshing problems can be avoided by discretizing the full scaffold into RVEs. To apply the direct CFD approach on the full-scale bioreactor system that contains a scaffold with an irregular pore geometry, it is necessary to run the CFD models on a HPC cluster (Jungreuthmayer et al. 2009; Stops et al. 2010). For example, Santamaría et al. (2013) enabled the simulation of the micro-fluidic environment within a small-sized Poly (L-Lactic Acid) scaffold (diameter = 3 mm, height = 1 mm) by applying a direct CFD approach using a cluster with 16 nodes (32 GB memory for each node) and 16 processors. However, with our multiscale CFD approach, simulating the micro-fluidic environment within the scaffold with larger size and highly irregular geometry is feasible even on a normal desktop computer/laptop. Therefore, this study will provide a useful computational framework in particular for the researchers who aim to simulate the fluidic environment within complex structures within short time but have limited access to the HPC facilities.

## Conclusion

In this study, a multiscale CFD model has been developed for simulating the micro-fluidic environment within a tissue engineering scaffold with highly irregular scaffold pore geometries. This multiscale CFD approach is demonstrated with reasonable accuracy. Specifically, percent error between the multiscale CFD-derived WSS and direct CFD-derived WSS is less than 10.5%, and the Pearson correlation coefficient between this multiscale CFD and direct CFD approaches is 0.86 on the resultant WSS distribution. Importantly, this multiscale CFD approach requires low-computational cost, and can be applied to model realistic-sized and scaffolds with irregular pore geometries regardless of HPC facilities limits. Therefore, it will be a useful tool for simulating the micro-fluidic environment within complex structures, e.g., tissue engineering scaffolds with highly irregular pore geometry under medium perfusion.

## Figures and Tables

**Fig. 11:**
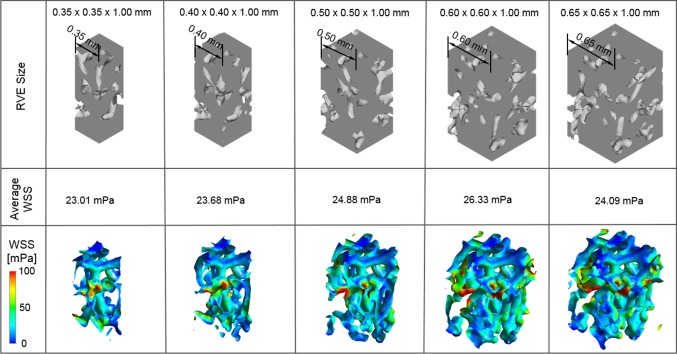
WSS within RVEs that have different sizes

**Fig. 12:**
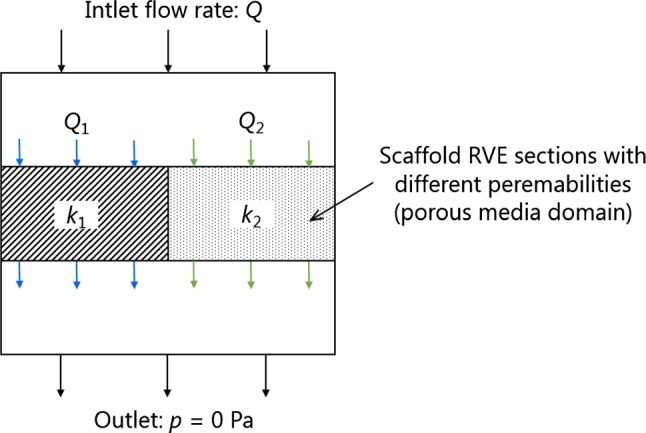
Idealized scaffold, which consists of 2 RVE sections with different permeabilities

**Fig. 13:**
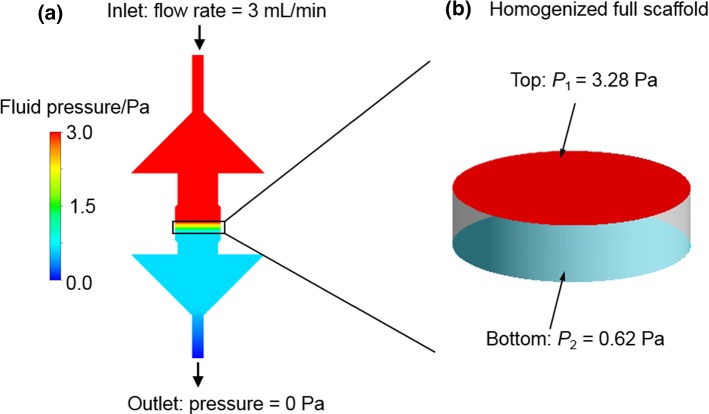
Fluid pressure distribution within **a** the perfusion bioreactor system and **b** the homogenized scaffold domain under a flow rate of 3 mL/min

## Appendix 1: Mesh size sensitivity analysis

The mesh sensitivity analysis was conducted on the RVE with 5 different mesh densities as listed in Table 1. As presented in Sect. 2.1, we meshed the fluid domain by tetrahedral elements (element type: FC3D4) with 5 different size types. In each mesh type, the actual mesh size was controlled by the local curvature of the scaffold surfaces with a maximum deviation factor (ratio between chordal deviation and element size) of 0.1. The boundary and loading conditions were kept the same as those presented in Sect. 2.3. Under the pressure drop of 0.7 Pa, the average WSS within RVE with different mesh densities was calculated, and results are shown in Table 1. It was found that no distinct influence on the resultant average WSS by mesh density. For instance, the variation of average WSS was within 3.2% among 5 different meshes (Table 1).

**Table 1:** Mesh sensitivity analysis: resultant average WSS within RVE scaffold influenced by different mesh densities

Mesh size (µm)	Element number	Average WSS (mPa)
10–1.0	1326,002	25.15
25–2.5	178,488	24.34
50–5.0	112,683	24.88
75–7.5	101,367	24.74

## Appendix 2: RVE size sensitivity analysis

We also examined the influence of RVE size on the resultant WSS in RVE. In this study, CFD approach was used for computing the WSS within 5 RVEs with different sizes (Fig. 11). In CFD model, the boundary and loading conditions were the same as those presented in Sect. 2.3. Specifically, a pressure drop of 0.7 Pa was prescribed over the scaffold (from top surface to the bottom surface). Four side surfaces were defined as symmetric boundaries, following Eq. (5). The scaffold surfaces were defined as non-slip wall, on which the WSS is calculated according to Eq. (6). The WSS distribution within each RVE is shown in Fig. 11. According to the results of direct CFD model (Fig. 6b), the average WSS within the whole scaffold was 24.00 mPa. Finally, it was found that the RVE size did not have profound influence on the resultant average WSS, i.e., maximum deviation < 9.7% among these 5 RVE sizes.

## Appendix 3: Analysis of scaffold homogenization

In Fig. 12, the shaded areas represent two scaffold sections with different permeability *k*_1_ and *k*_2_, thus representing an inhomogeneous scaffold. These two sections had a cross-sectional area *A*_1_ and *A*_2_, respectively. At the inlet, a flow rate of *Q* was applied. Assumptions were: (1) the fluid pressure *p* was constant throughout the top compartment (and zero throughout the bottom compartment); (2) no fluid flow between the compartments (lateral flow) occurred; (3) the viscosity was constant.

According to Darcy’s law, the total flow rate through the scaffold (*Q*) then was:

7 $$\begin{aligned} Q = & Q_{1} + Q_{2} \\ = & \frac{{\Delta p \cdot k_{1} \cdot A_{1} }}{\mu \cdot L} + \frac{{\Delta p \cdot k_{2} \cdot A_{2} }}{\mu \cdot L} \\ = & \frac{{\Delta p \cdot \left( {k_{1} \cdot A_{1} + k_{2} \cdot A_{2} } \right)}}{\mu \cdot L} \\ = & \frac{{\Delta p \cdot \left( {k_{1} \cdot f_{1} + k_{2} \cdot f_{2} } \right)A}}{\mu \cdot L} \\ \end{aligned}$$

where, *f*_1_ and *f*_2_ are the volume fractions of Sects. 1 and 2, respectively, and *A* is the total cross-sectional area.

The pressure drop then was expressed by reorganizing the equation as:

8 $$\Delta p = \frac{Q \cdot \mu \cdot L}{{\left( {k_{1} \cdot f_{1} + k_{2} \cdot f_{2} } \right)A}} .$$

As was clear from above equations, the same pressure drop would be obtained from a homogeneous scaffold with a permeability *k* that met the condition:

9 $$k = k_{1} \cdot f_{1} + k_{2} \cdot f_{2} .$$

If the two domains had the same volume fraction (i.e., *A*_1_ = *A*_2_ in Fig. 12), Eq. (9) was reduced to simply averaging of the permeabilities of the individual domains:

10 $$k = \frac{{k_{1} + k_{2} }}{2}$$

In our example (Fig. 9), all RVE sections had the same cross-sectional area and the corresponding homogeneous permeability. Therefore, the permeability of whole scaffold could be calculated by simply averaging the 9 individual RVE permeabilities (Fig. 9).

## Appendix 4: Pressure results from macro-model of perfusion bioreactor

Having solved the macro-model of the perfusion bioreactor, the fluid pressure distribution within the whole bioreactor was obtained (Fig. 13a). Within the scaffold, a pressure drop of 2.66 Pa (3.28–0.62 Pa from top to bottom surface) was calculated (Fig. 13b). This pressure drop would be used for defining the loading conditions of micro-CFD model in Sect. 2.4 and 3.2.
